# *Acanthamoeba castellanii* is not be an adequate model to study human adenovirus interactions with macrophagic cells

**DOI:** 10.1371/journal.pone.0178629

**Published:** 2017-06-07

**Authors:** Elodie Maisonneuve, Estelle Cateau, Nicolas Leveque, Sihem Kaaki, Agnès Beby-Defaux, Marie-Hélène Rodier

**Affiliations:** 1 Laboratoire Ecologie et Biologie des Interactions, Equipe Microbiologie de l’Eau, UMR CNRS 7267, Université de Poitiers, Poitiers, France; 2 Laboratoire de parasitologie et mycologie médicale, CHU La Milètrie, Poitiers, France; 3 Laboratoire de virologie et mycobactériologie, CHU La Milètrie, Poitiers, France; 4 Laboratoire Inflammation, Tissus Epithéliaux et Cytokines, EA 4331, Université de Poitiers, Poitiers, France; 5 Unité de pathologie ultrastructurale et expérimentale, Laboratoire d’anatomie et cytologie pathologiques, CHU la Milètrie, Poitiers, France; Swedish Neuroscience Institute, UNITED STATES

## Abstract

Free living amoebae (FLA) including *Acanthamoeba castellanii*, are protozoa that feed on different microorganisms including viruses. These microorganisms show remarkable similarities with macrophages in cellular structures, physiology or ability to phagocyte preys, and some authors have therefore wondered whether *Acanthamoeba* and macrophages are evolutionary related. It has been considered that this amoeba may be an *in vitro* model to investigate relationships between pathogens and macrophagic cells. So, we intended in this study to compare the interactions between a human adenovirus strain and *A*. *castellanii* or THP-1 macrophagic cells. The results of molecular and microscopy techniques following co-cultures experiments have shown that the presence of the adenovirus decreased the viability of macrophages, while it has no effect on amoebic viability. On another hand, the viral replication occurred only in macrophages. These results showed that this amoebal model is not relevant to explore the relationships between adenoviruses and macrophages in *in vitro* experiments.

## Introduction

Adenovirus are viruses infecting a broad range of hosts from birds to fish. Human adenovirus (HAdV) have been isolated for the first time by Rowe in 1953, in a sample of amygdala and respiratory secretions [1]. They are non-enveloped viruses, constituted of an icosahedral capsid enclosing a double-stranded DNA genome of approximately 36,000 base pairs [2]. Resistant in external environment due to the absence of envelope, HAdV can be transmitted *via* the feco-oral route with a direct or indirect transmission, when the water is contaminated [3]. HAdV can thus be used to monitor the foecal contamination, so the virological quality of water [4]. They are also responsible for a wide range of human pathologies according to their serotype. For example, HAdV41 detected in wastewater and surface water is a leading cause of childhood gastroenteritis [4]. Pathologies also commonly encountered are respiratory infections as well as eye infections [5–7]. In order to replicate, HAdV need a host cell in which the viral cycle may occur, generally leading to cell lysis.

In hydric environment, HAdV are mixed with other microorganisms as Free-Living Amoebae (FLA). FLA are ubiquitous protozoa, capable of feeding and prey bacteria, fungi, viruses or protozoa [8–11]. Among the environmental FLA, the genus *Acanthamoeba* is particularly represented. These amoebae are known for their capacity to serve as a reservoir for multiplication but also for the dissemination of some pathogenic bacteria and viruses [10]. Among amoeba-resistant viruses, Mimivirus is a giant virus that causes respiratory infections and is able to develop and multiply in FLA of the genus *Acanthamoeba* [12].

However, the relationships between FLA and HAdV are not well known. In 2007, Lorenzo-Morales showed the presence of HAdV in *Acanthamoeba* strains genotype T3, T4, et T7 [13]. More recently, it has been shown that HAdV do not replicate in *Acanthamoeba* despite their presence in the amoeba cytoplasm [14]. Overall, the role of FLA in HAdV persistence in hydric environment should be further clarified.

Given their ability to phagocyte microorganisms and the fact that some pathogens can resist to destruction by FLA, these protozoa have been regularly compared to macrophages [15,16] which are professional phagocytic cells participating in innate immunity and whose main role is to phagocyte microorganisms as well as cellular debris. Some authors have therefore wondered whether this amoeba may represent an *in vitro* model to investigate relationships between pathogens and macrophagic cells.

In this context, the first objective of this study was to assess permissivity of *A*. *castellanii* to entry and replication of a HAdV strain isolated from a clinical sample. The second objective of our work was then to compare the biology of HAdV in *A*. *castellanii* with that in THP-1 macrophagic cells to investigate the use of *Acanthamoeba castellanii* as a relevant model of study of interplay between pathogens and macrophagic cells.

## Materials and methods

### Organisms and culture media

The Adenovirus serotype B3 strain used in this study has been isolated from an eye swab of a patient suffering from keratitis and serotyped by serum neutralization assay (French National Reference Center for enterovirus and parechovirus, Lyon, France). The virus was propagated and titrated with the TCID50 assay protocol on HEp-2 cells in DMEM medium supplemented with 10% heat-inactivated fetal bovine serum, 1% streptomycin and 1% penicillin at 37°C in the presence of 5% CO_2_ for 5 days.

*A*. *castellanii* ATCC 30234 was grown in 150 cm^2^ tissue culture flasks in PYG broth at 27°C [17]. When cells formed a monolayer, the trophozoites were harvested by tapping the flasks and washed three times in Page’s modified Neff’s amoeba saline (PAS, containing in 1 L of distilled water, 120 mg NaCl, 4 mg MgSO_4_.7H_2_O, 4 mg CaCl_2_.2H_2_O, 142 mg Na_2_HPO_4_ and 36 mg KH_2_PO_4_).

THP-1 monocytic cell line was maintained in RPMI-1640 supplemented with 10% heat-inactivated fetal bovine serum, 1% streptomycin and 1% penicillin at 37°C in the presence of 5% CO_2_. THP-1 cells were differentiated for 72h with 50 ng/mL of phorbol-12-myristate-13-acetate (PMA) in fresh RPMI 1640 medium with 10% FBS. Before use, the macrophages were washed twice with RPMI 1640 medium in order to remove PMA.

### Co-culture experiments

Twenty four-well plates were seeded with one μL of the amoebic trophozoite or macrophage suspension (containing 1.10^5^ cells) in PAS containing 10% RPMI (PAS-RPMI). The amoebic trophozoites and the macrophages were allowed to adhere to the wells for 2 hours at 27°C or 37°C for amoebae or at 37°C under 5% CO_2_ for macrophages. A hundred μL of the viral suspension at 1.105 TCID 50/mL were added in each well (Multiplicity Of Infection of 0.1). Incubation was then carried out during 24, 48, 72 and 96 h at 27°C or 37°C for amoebae or at 37°C under 5% CO_2_ for macrophages. In order to evaluate the evolution of the virus concentration without amoebae or macrophages, non replicative negative controls have been carried out in incubating viruses alone at 27°C and 37°C in PAS-RPMI. Microscopical examination of the co-cultures using trypan blue (0.1mg/mL) staining was also carried out in order to determine the viability of the phagocytic cells.

All experiments have been reproduced three times, each time in triplicate.

### qPCR assays

Replication of viral DNA was monitored in each co-culture experiment and in each control at 24, 48, 72 and 96 h post-infection by real-time quantitative PCR assay. Total DNA was extracted separately from 200μL of the cell supernatant and from the cell pellet obtained after centrifugation at 500g during 7 min of the amoebic trophozoite or macrophage suspension. Extraction was performed with the Qiagen kit QIAamp^®^ DNA mini and extracted DNA was recovered in 50μL of elution buffer. One hundred nanograms of DNA were then subjected to the real-time qPCR using the ADENOVIRUS ELITe MGB^™^ kit according to the manufacturer’s instructions. The viral load was expressed as the number of virus genome copies either per mL of cell supernatant or per microgram of extracted DNA if measured in the cell pellet.

### Immunofluorescence experiments

At each point of the time course of amoebic trophozoite or macrophage infection, the intracellular synthesis of viral antigens was monitored by direct immunofluorescence. Co-cultures of virus and phagocytic cells or mock-infected phagocytic cells were washed three times in PAS and the supernatant was finally removed. The obtained pellet was then seeded onto slides that were dried. Macrophages cells were fixed in cold acetone for 10 min and amoebic trophozoites in cold methanol for 15 min, then 10 min in triton 0,2% to permeabilize the membrane cells. After drying, the slides were treated for 15 min at 37°C in wet room with a monoclonal anti-adenovirus group FITC antibody (Argene, bioMerieux), washed with PBS and the cells were counterstained in red with Evans blue 0,1% in order to improve the visualization of viral antigens in green. Samples were then monted in oil and examined with a fluorescence microscope with a 40x lens objective.

### Electron microscopy

The potential internalization of adenovirus by phagocytic cells was also investigated by electron microscopy. Samples after 24 or 72 h of co-cultures were fixed for 1 h in phosphate buffer 0.1 M, containing 4% glutaraldehyde at 4°C. Cells were washed four times in PBS and post-fixed with 1% OsO_4_ in phosphate buffer 0.1 M for 1 h at 4°C. The samples were dehydrated in an acetone series and embedded in araldite resin. Sections were stained with uranyl acetate and lead nitrate before examination with a Jeol 1010 transmission electron microscope.

### Statistical analysis

Statistical analyses were performed using the Mann-Whitney test. P<0.05 was considered statistically significant. The analysis was performed using Prism5 for PC (GraphPad Software).

## Results

### Viability of phagocytes in co-culture with HAdV B3

**Fig 1:**
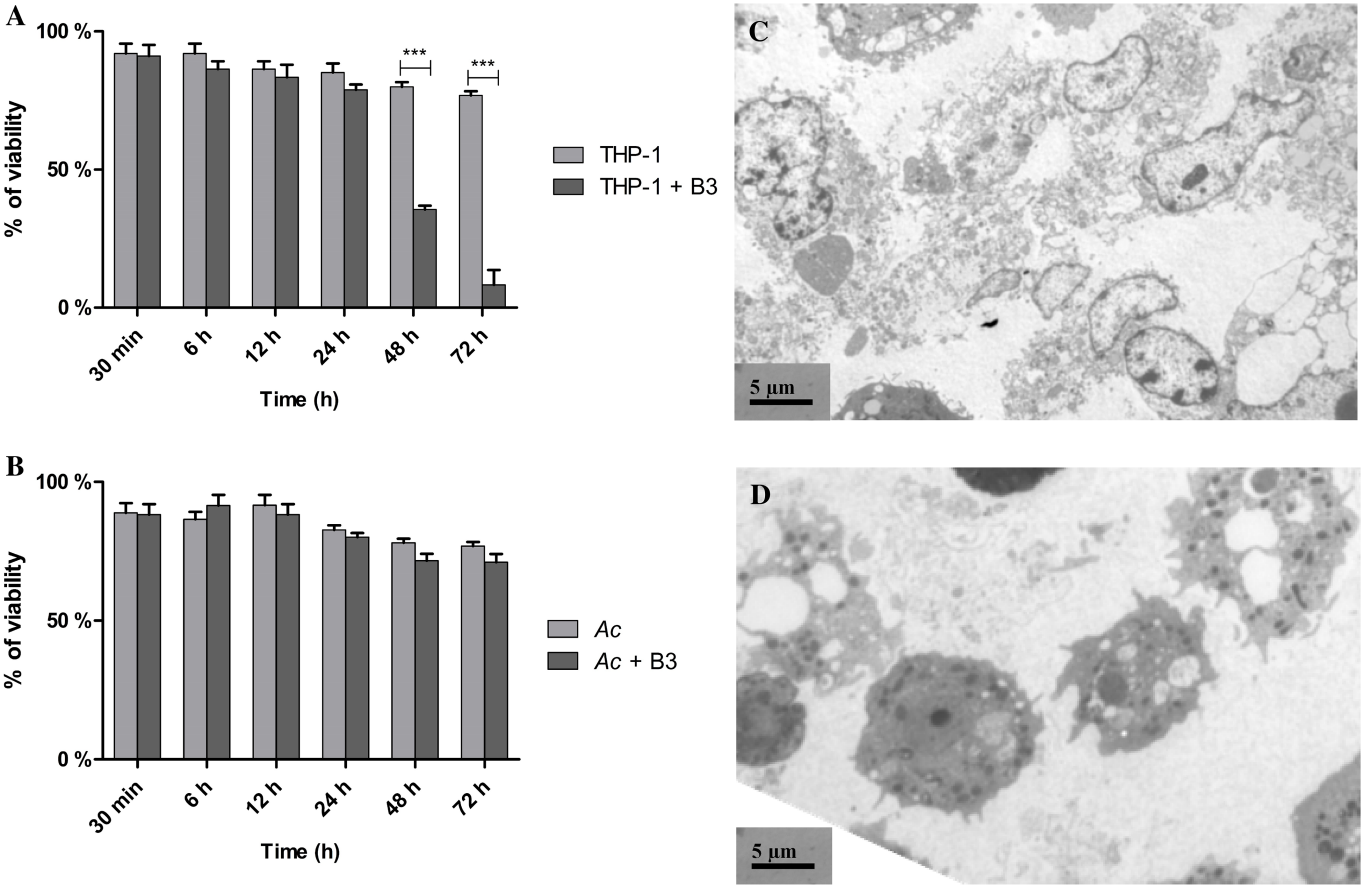
Influence of adenovirus on phagocytic cell viability after 3h, 6h, 24h, 48h and 72h of coculture. (A) macrophages or (B) *A*. *castellanii*. Ac: *A*. *castellanii* control, *Ac +* B3: Coincubation *A*. *castellanii*/adenovirus B3, THP-1: THP-1 control, THP-1 + B3: Coincubation THP-1/Adenovirus B3. All values correspond to mean of nine replicates. Values with stars are significantly different (p<0.001 for ***). Similar results were obtained after incubation of the co-culture *A*. *castellanii* / HAdV B3 at 37°C.

The viability of amoebae and macrophages was checked using trypan blue after incubation with HAdV B3, using a glasstic disposable slide with quantitative grid (Kova^®^). The results showed that the viability of the THP-1 cells incubated with the virus began to decrease after 48h of co-culture, in comparison with THP-1 alone (Fig 1A and 1C). At the opposite, there was no effect of the virus on *A*. *castellanii* viability during the time of experimentation (Fig 1B and 1D) whatever the temperature of incubation.

Transmission electron microscopy images of (C) Macrophages THP-1 and (D) *A*. *castellanii* trophozoïtes infected by Adenovirus serotype B3 after 72 h of co-culture. Scalebar 5 μm.

### Quantitative evaluation of viral DNA

**Fig 2:**
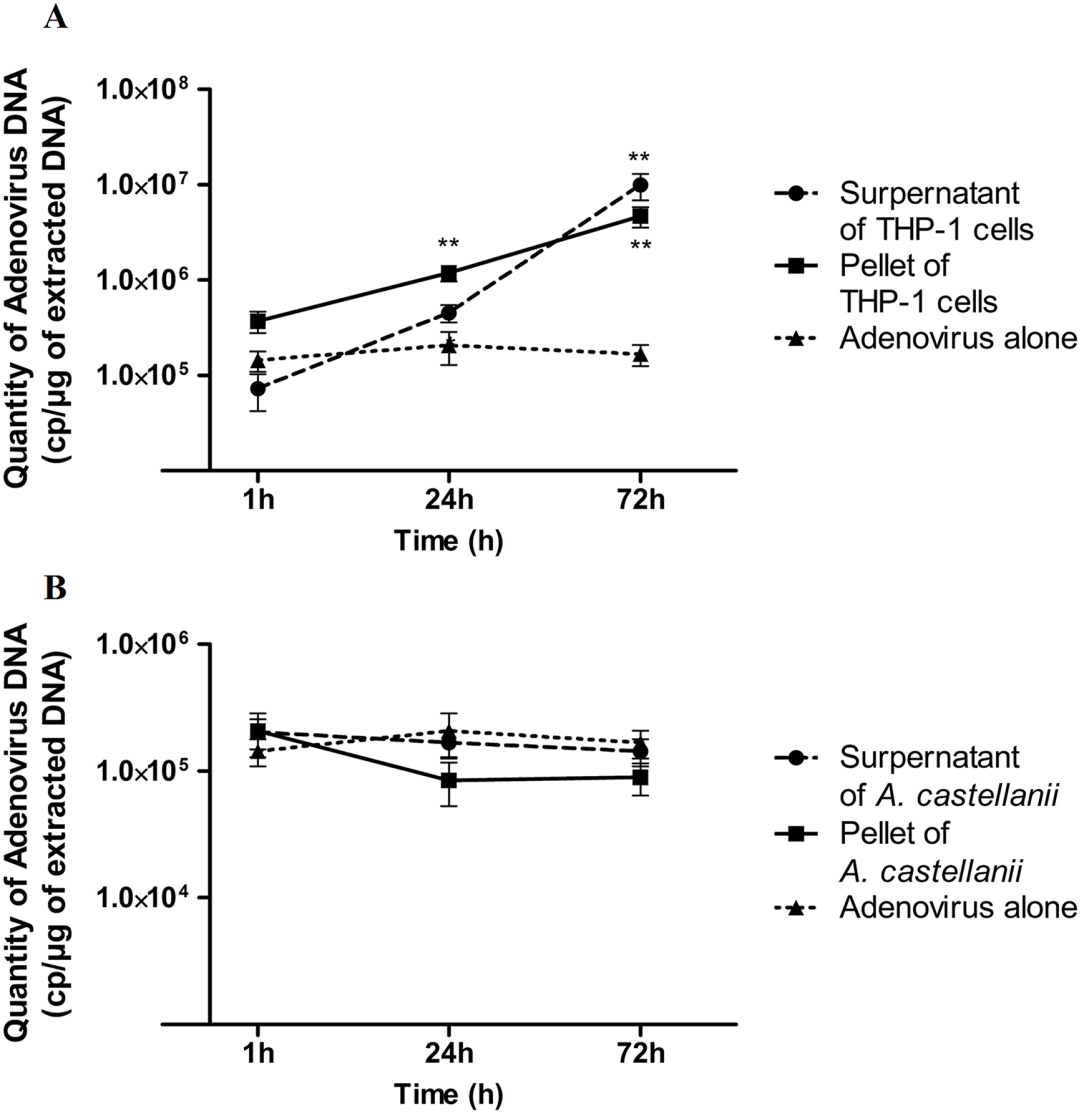
Quantification of adenovirus serotype B3 in supernatant or pellet of macrophage THP-1 (A) or *A*. *castellanii* (B). All values correspond to mean of six replicates. Values with stars are significantly different (p < 0.05 for * and p < 0.01 for ** Mann-Whitney test). Similar results were obtained after incubation of the co-culture *A*. *castellanii* / HAdV B3 at 37°C.

The supernatants and the pellets of the viral co-cultures conducted with the two phagocytic cells were recovered after the different times of incubation. The viral DNA was then extracted and quantified by qPCR. The results obtained from the viral co-culture with macrophages THP-1 showed that the amount of HAdV B3 DNA increased during the 72 h of the experiment, in the culture supernatant as well as in the pellet of macrophagic cells (Fig 2A). On the contrary, the presence of the amoeba *A*. *castellanii* had no effect on the viral load, at 27°C or at 37°C. Indeed, the amount of viral DNA remained stable during the 72 h of the assay, either in the co-culture supernatant or in the amoebae pellet (Fig 2B).

### Immunofluorescence observation

**Fig 3:**
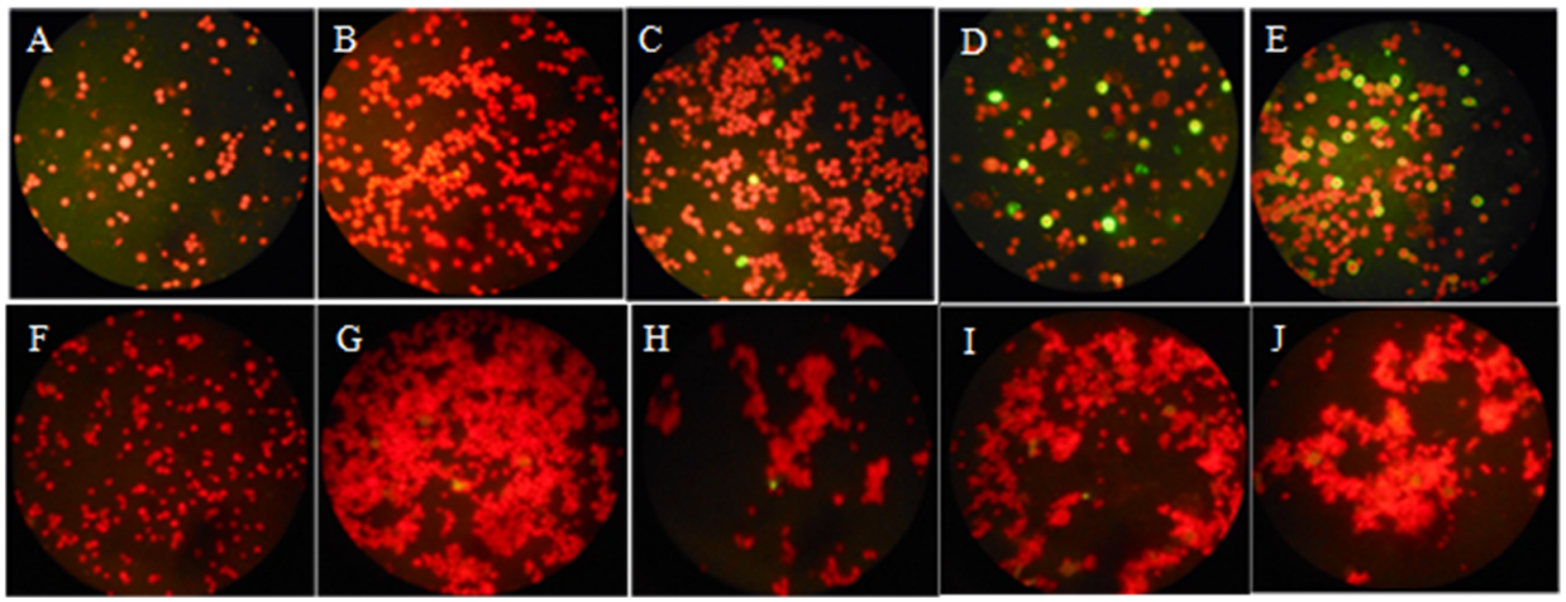
Immunofluorescence results (A-E) macrophages THP-1, (F-J) *A*. *castellanii*, (A,F) mock-infected, (B,G) 1h, (C,H)24h, (D,I) 48h, and (E,J) 72h post-infection. Adenovirus antigens are stained in green in phagocytics cells counterstained in red.

The presence of HAdV B3 into the two phagocytic cells was tested by a direct immunofluorescence assay. The microscopic observations showed an increase of virus antigens in macrophages over the time at 24, 48 and 72 h (Fig 3C to 3E), whereas no virus antigen was visible in the cells at the beginning of the experiment (Fig 3B). However, during co-culture assays with *A*. *castellanii*, a few viruses were internalized as early as 24 hours, but afterthat, there was no increase in fluorescence over time (Fig 3G to 3J). These results suggest that HAdV B3 is internalized by THP-1 cell as well as by *A*. *castellanii*, but only multiply in THP-1 cells. These results are quite in agreement with qPCR results that also showed the multiplication of HAdV B3 only in macrophages THP-1.

### Electron microscopy

**Fig 4:**
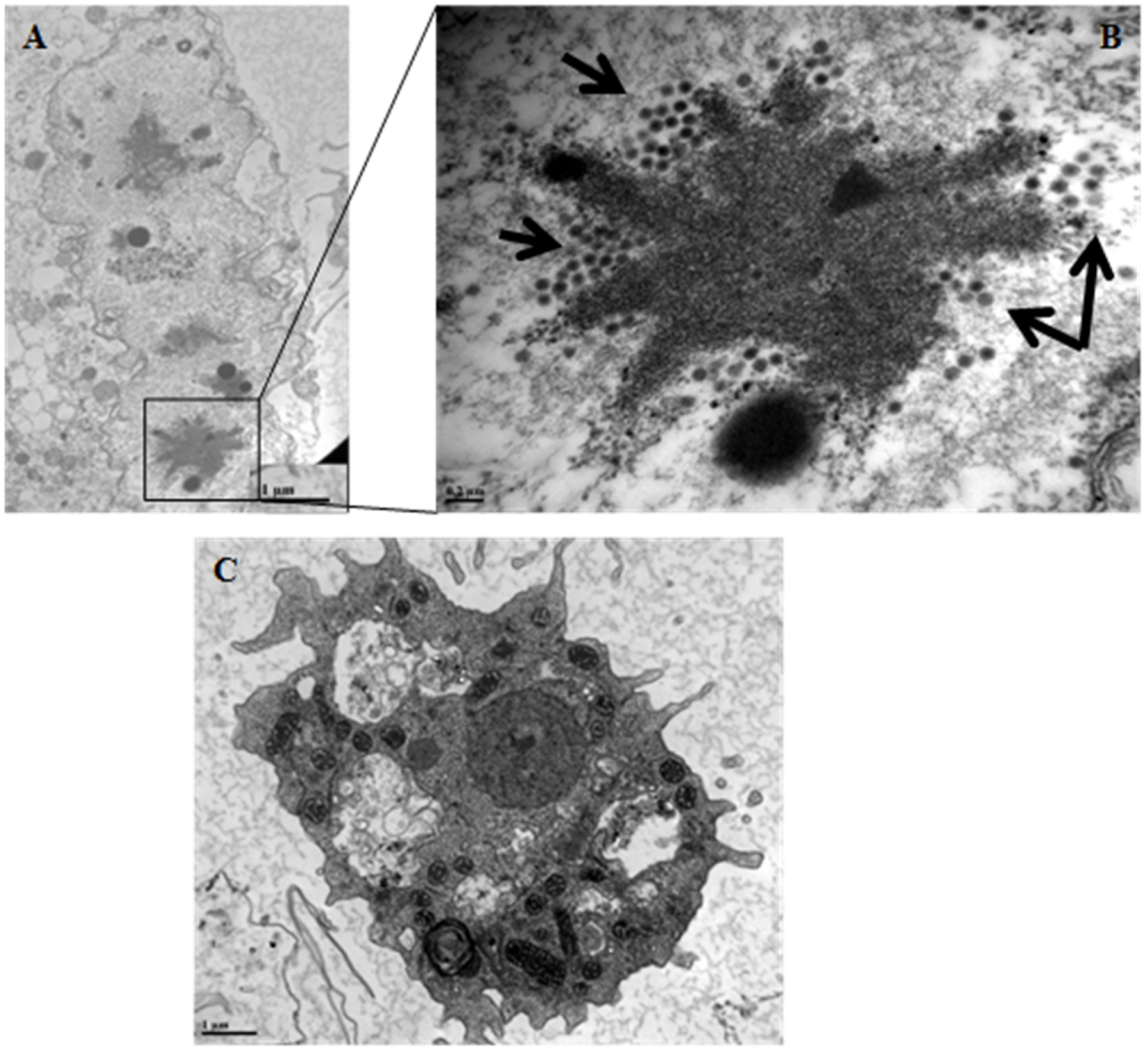
Transmission electron microscopy images of (A, B) macrophages THP-1 and (C) *A*. *castellanii* trophozoïtes infected by adenovirus serotype B3 after 72 h of co-culture adenovirus elements are indicated by arrows. Scalebar 1 μm A, C and 0,2 μm B.

The first observation of the co-cultures by electron microscopy after 24 h showed the absence of adenovirus inside the two phagocytic cells: *A*. *castellanii* or macrophages (data not shown). However, after 72 h of co-culture, viral particles were observed inside THP-1 cells likely reflecting virus replication (Fig 4A and 4B). Conversely, after 72 h of co-culture with *A*. *castellanii*, no virion was found inside the amoebae (Fig 4C). These observations were also in agreement with the molecular data and immunofluorescence observations.

## Discussion

*Acanthamoeba* is a genus found in the biosphere, frequently in soil and natural or man made aquatic environments [18,19]. These FLA feed on bacteria, algae, fungi, protozoa, viruses or other organic particles [9,10]. It has been described, for some bacteria, an adaptation to the protozoan niche with survival and sometimes multiplication of ingested bacteria with or without amoeba lysis, leading to bacterial protection and colonization of other environments [20,21]. Since FLA and viruses can be found in the same environments, some authors have also investigated the potential interactions between these microorganisms in order to determine if FLA could play a role in the viral multiplication and spread. Most of the studies have recently concerned the so called “giant viruses” like Mimiviruses or Pandoraviruses which are endocytobionts within FLA [22]. Besides the giant viruses, a few studies have been conducted with other viruses, in particular those infecting humans. As human noroviruses (HuNoVs) are involved in virus-related waterborne disease outbreaks, Huesh et al have studied their interactions with *Acanthamoeba spp*. and demonstrated that *A*. *castellanii* is able to interact with these enteric viruses [23]. After adhesion, HuNoV is internalized in *A*. *castellanii* trophozoites that could therefore play the role of viral reservoir in water systems. HAdV, for their part, are responsible for 24% of the virus-related waterborne outbreaks [24]. They have been detected in various water environment worldwide [25] where they can survive longer than other nonenveloped viruses such as enteroviruses [26]. HAdVs were found to be one of the most promising human specific viral markers in microbial source tracking [27]. Lorenzo-Morales et al. have reported that four different HAdV serotypes (HAdV-1, 2, 8, and 37) have been detected in *Acanthamoeba* strains from water sources in the Canary islands reflecting human contamination [13]. More recently, HAdV genome has also been detected and quantified in 62% of *Acanthamoeba* isolates from brasilian swimming pools. In this study, Scheid et al. demonstrated the intake of HAdV serotypes 11 and 41 by different *Acanthamoeba* strains, which can therefore act as carriers or vectors of HAdV in the same environment [14]. Moreover, according to Verani et al, *Acanthamoeba polyphaga* can even protect HAdVs from sodium hypochlorite water disinfection treatment [28]. In all these studies, even it can be proved that HAdVs can survive inside FLA, no viral replication within these protozoa has been demonstrated. This is also the case in the present work where we demonstrated by molecular methods that HAdV DNA can persist in or attached to *A*. *castellanii* during co-culture experiments, but without increase of the viral DNA load and thus without viral replication. Moreover, immunofluorescence assay using specific anti-HAdV antibodies allowed us to find rare viral antigen expression in amoebae, which do not influence the viability of their host. However, only not “infected” *A*. *castellanii* trophozoite has been observed using electronic microscopy, probably because of the low sensitivity of this method. Finally, from our experiments and those of others, we can conclude that if *A*. *castellanii* can internalize HadV by expressing yet-unknown receptors on its membrane able to interact with viral capsid, thisprotozoa does not have the cellular machinery necessary for HAdV replication. *A*. *castellanii* can consequently act only as a carrier but not as a booster of the viral load in the environment.

Macrophages are described as “professional” phagocytic cells which express receptors on their surfaces that detect signals normally not found in healthy tissues. They have a central role in protecting the host by destroying the pathogens after their internalisation through the merger between phagosomes and lysosomes [29]. Macrophages play a significant role in the innate immune response to HAdV *in vivo*, although the mecanism by which they might internalise these viruses are not well known [30]. Given that phagocytosis is specified for particles typically larger than 0.5–1 mm, viruses smaller than 0.5 mm are internalised by endocytosis rather than phagocytosis. HAdV are quickly taken up and internalised into macrophages but 100-1000-fold less than into epithelial cells that express the cell surface receptor CAR (coxsackievirus adenovirus receptor). The internalisation of HAdV in macrophages could require a cell surface integrin and be modulated by the availability of low-affinity, high avidity receptors, such as scavenger receptors [31,32]. Moreover, macrophages have been shown to be permissive to HAdV replication *in vitro* [33]. Some data also reported that HAdV can persistently infect monocytes *in vivo*, providing a reservoir for latent infection [34]. Finally, Di Paolo et al. presented data demonstrating that, after interaction with the virus, Küpffer cells undergo rapid death [35]. In this report, we showed by molecular and microscopical methods that HAdVs are internalized and replicate in THP-1 cells, leading to the decrease of the macrophages viability.

Some authors have previously compared *Acanthamoeba* to human macrophages in some structures or behavior, like surface receptors or phagocytic activity [36,37]. However, we demonstrated that, in our experimental conditions, interactions between HAdV and macrophages are quite different from interactions between HAdV and *A*. *castellanii*. In conclusion, the *A*. *castellanii* model do not seem to be relevant to explore the relationships between HAdV and immune cells like macrophages in *in vitro* experiments.
